# Skin Hyperpigmentation Due to Post-Surgical Adrenal Insufficiency Regressed with the Dexamethasone Treatment

**DOI:** 10.3390/jcm11185379

**Published:** 2022-09-13

**Authors:** Tungalagtamir Shagjaa, Viola Sanga, Gian Paolo Rossi

**Affiliations:** 1 PhD Arterial Hypertension and Vascular Biology, Department of Medicine—DIMED, University of Padua, 35128 Padua, Italy; 2 Department of Neurology, Mongolian National University of Medical Sciences—MNUMS, Ulaanbaatar 14210, Mongolia; 3 Hypertension and Emergency Unit, Department of Medicine—DIMED, University of Padua, 35128 Padua, Italy

**Keywords:** adrenalectomy, adrenal insufficiency, dexamethasone, glucocorticoid replacement therapy, primary aldosteronism

## Abstract

Primary adrenal insufficiency (AI) due to bilateral adrenalectomy is not uncommon and causes skin hyperpigmentation, which worsens quality of life. Case description: A 50-year-old lady presented with skin hyperpigmentation after spare adrenalectomy for recurrent primary aldosteronism. In 2002 she has her first unilateral adrenalectomy and was cured at follow-up. After 16 years she developed primary aldosteronism, which was treated by spare adrenalectomy. She thereafter developed AI and started glucocorticoid replacing therapy, which did not prevent the development of full-blown skin hyperpigmentation. The addition of a low dose of dexamethasone (0.5 mg/day) to the ongoing adrenal replacement therapy normalized her plasma adrenocorticotropic hormone (ACTH) levels and regressed skin hyperpigmentation without causing Cushing-like symptoms or signs. Conclusions: This clinical case provides compelling evidence for a place for low-dose dexamethasone for regressing skin pigmentation in patients with primary AI.

## 1. Introduction

With an incidence of 4.4–6 cases per million per year [1], primary adrenal insufficiency (AI) is not uncommon. Although epidemiological data are lacking, owing to the increased awareness and the expanding number of adrenalectomies for primary aldosteronism (PA), Cushing’s syndrome, and pheochromocytoma, and the treatment of these conditions sometimes with bilateral adrenalectomy, the likely incidence of post-surgical AI will increase in the next decades. The Endocrine Society has devoted comprehensive practical guidelines for the management of AI [2]. However, no recommendations are provided for the prevention and regression of skin hyperpigmentation, which occurs as a result of the loss of glucocorticoid negative feedback on POMC-ACTH-melanocortin. Skin hyperpigmentation can be troublesome for many patients. We herein report a case of primary AI with overt skin hyperpigmentation that developed soon after bilateral adrenalectomy for PA. A glucocorticoid replacement therapy focused on blunting the hypothalamic-pituitary axis (HPA) by addition of low-dose dexamethasone, which completely regressed skin hyperpigmentation within a few months without causing any symptoms or signs of AI or Cushing’s syndrome.

## 2. Case Description

A 50 year-old Caucasian lady was hospitalized in 2002 for a fever of unknown cause. An abdominal computed tomography (CT) led to the incidental discovery of a 20 mm nodule in the left side adrenal gland. At that time, she had a three year history of high blood pressure (BP) and, therefore, she was screened for PA. Hypokalemia (3.3 mmol/L), a suppressed plasma renin activity (PRA < 0.20 ng/mL/h) and an inappropriately high plasma aldosterone concentration (PAC 10 ng/dL) with a raised aldosterone-to-renin-ratio (ARR) 50 (ng/dL)/(ng/mL/h)) confirmed the diagnosis. A bilaterally successful adrenal vein sampling (AVS) showed a high lateralization index (LI) of 82 (cut-off value > 2.0) on the right side. Right laparoscopic adrenalectomy was performed uneventfully, and cortical nodular hyperplasia was found at pathology examination in the resected adrenal gland.

During the following seven years she showed normal BP values on no drugs, serum potassium levels, aldosterone, and renin values were consistently normal at yearly follow-up checks. However, in 2016, 13 years after adrenalectomy, her direct renin concentration (DRC) showed very low values (<2.0 mIU/L), albeit with a normal PAC value (4.9 ng/dL).

From 2018 her systolic and diastolic BP values (173 ± 15/91 ± 11 mmHg) were elevated, and borderline serum potassium values (3.7 mmol/L) were noted. Furthermore, she showed an increase of baseline ARR [65 (ng/dL)/(ng/mL/h)]. A new AVS showed a markedly elevated relative aldosterone secretion index (RASI) [3] (PACside/PCCside)/(PACivc/PCCivc) of 57 (normal value < 0.90), which confirmed left adrenal hypersecretion of aldosterone. An abdominal CT demonstrated a mass of 32 mm in the remaining left adrenal gland. Partial (spare) adrenalectomy was, therefore, performed uneventfully in May 2020.

**Figure 1:**
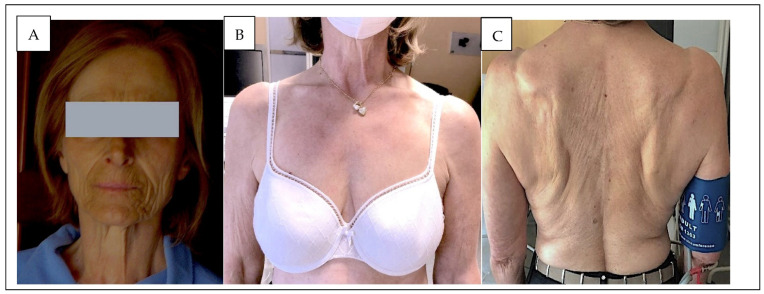
(**A**) The skin pigmentation with adrenal replacement treatment. (**B**,**C**) Skin pigmentation regression after treatment with dexamethasone in addition to adrenal replacement therapy.

After surgery, BP (<130/85 mmHg), serum potassium, and ARR values were consistently normal, even though renin remained low. Moreover, she developed episodes of hypotension, and her morning serum cortisol value was depleted, ranging from 11 to 14 nmol/L (normal value 138–635 nmol/L). Replacement treatment with cortisone acetate 37.5/day was started by her endocrine surgeon, but six months later, while on such replacement therapy, she developed progressive skin pigmentation, which involved her body (Figure 1A) and entire face, which she considered very disturbing. Laboratory findings showed a permanent increase of morning adrenocorticotropic hormone (ACTH) to 999 ng/L, i.e., about 20 fold above the upper limit of the normal range (normal value: <50 ng/L). In the meantime, skin pigmentation had worsened and was judged to be unbearable, while her PRA was normal 1.59 ng/mL/h (range from 0.6 to 4.3 ng/mL/h).

Based on the hypothesis that dexamethasone treatment, by preventing proopiomelanocortin (POMC) production, could reduce her skin pigmentation, we added 0.5 mg dexamethasone to the ongoing adrenal replacing treatment. After six months of treatment, skin hyperpigmentation vanished (Figure 1B,C) and (ACTH) values dropped to 11.6 ng/L. At 12 months follow-up, she was normotensive (BP values 137/84 mmHg), and no symptoms or signs of AI or skin hyperpigmentation were noticed, and her renin value, serum potassium, and sodium levels were normal.

At 18 months follow-up the patient showed no signs whatsoever of glucocorticoid-related side effects including weight gain, type 2 diabetes mellitus, osteoporosis or signs of Cushing’s syndrome.

## 3. Discussion

This case provides proof of the concept that the addition of a tiny dose of dexamethasone to an effective replacement therapy of primary AI with cortisone acetate allowed the suppression of excess POMC release and, therefore, regression of skin hyperpigmentation. This observation is quite relevant because bilateral adrenalectomy might be necessary for the treatment of recurring Cushing’s disease and PA. The latter is a common cause of secondary hypertension as it involves between 5.9% of the hypertensive patients seen by general practitioners and over 20% of those with drug-resistant hypertension [4,5]. Unilateral laparoscopic adrenalectomy is the treatment of choice for PA, as it leads to complete biochemical cure in over 95% of the cases and to cure of high BP in over 45% when a unilateral form is identified by AVS [6,7]. However, recurrence of aldosteronism post-adrenalectomy after a long-term cure of PA has been reported, [8] suggesting that this case is not unique. Moreover, bilateral adrenalectomy can be necessary for patients with drug-resistant hypertension due to PA and familial hyperaldosteronism type III A [9,10].

Partial (spare) adrenalectomy has been largely abandoned in the treatment of the primary aldosteronism because of a high rate of recurrence, likely because of the multifocal nature of the disease. In this case, the first surgical treatment was followed by complete biochemical and surgical cure, thus proving unambiguously that the removed adrenal gland had been responsible for the PA. Upon recurrence of PA, after team discussion, we offered this lady partial (spare) adrenalectomy of the nodule, because the RASI [11] showed an unambiguous excess aldosterone production from the remnant adrenal gland and, moreover, she wished to achieve a long-term cure. We hoped that the remaining adrenal tissue could avoid overt AI. However, at follow-up soon after surgery, AI can be challenging as it leads to loss of glucocorticoid and mineralocorticoid function, which features hypotension, a rise of renin, hyponatremia, hyperkalemia, and metabolic acidosis with a normal anion gap. At variance with this prediction, the lady developed overt AI with skin hyperpigmentation (Figure 1A) in spite of the residual adrenal tissue. Although she had been alerted to this possible complication, this turned out to be a major discomfort for her.

Hence, based on the premise that dexamethasone is the most effective steroid in inhibiting POMC [12] as shown in Table 1, the drug was added at a low dose to the ongoing replacement glucocorticoid therapy to verify if it could regress skin hyperpigmentation. Results were impressive: within 6 months her skin hyperpigmentation had totally vanished (Figure 1B,C) and no sign of Cushing’s syndrome appeared at longer follow-up.

## 4. Conclusions

Current guidelines for AI recommend glucocorticoid therapy by hydrocortisone or cortisone acetate as the optimal treatment strategy, but they also warn against the usage of dexamethasone for the risk of Cushing-like side effects and the difficulty of dose titration [2]. Furthermore, Cassarino et al. [13] reported that dexamethasone had in vitro diverging effects on POMC expression in human corticotropin secreting adenomas. Nonetheless, even at small doses, dexamethasone being one of the most effective agents in blunting the HPA (Table 1), we hypothesized that it could effectively regress skin hyperpigmentation. In order to widen the recommendation, which is suggested by this proof-of-concept study, before using dexamethasone at low doses in all patients with adrenal insufficiency and skin hyperpigmentation, a population-based study with a long follow-up is necessary.

This prediction was fully verified: our clinical case provides compelling evidence in vivo for a beneficial effect of low-dose long-term dexamethasone for regressing skin pigmentation in patients with primary AI via decreased POMC and ACTH levels. Moreover, the potency of dexamethasone and its long biologic half-life [12] make it suitable for long-term once-a-day dosing in the chronic treatment of AI.

## Figures and Tables

**Table 1:** Glucocorticoid equivalences adapted with permission from Nicolaides, N.C. et al. [12]. Copyright year 2022, Copyright owner Kenneth Feingold.

Glucocorticoid	Equivalent Dose (mg)	HPA Suppression	Plasma Half-Life (min)	Biologic Half-Life (h)
Cortisol	20.0	1.0	90	8–12
Cortisone	25.0		80–118	8–12
Prednisone	5.0	4.0	60	18–36
Prednisolone	5.0		115–200	18–36
Triamcinolone	4.0	4.0	30	18–36
Methylprednisolone	4.0	4.0	180	18–36
Dexamethasone	0.75	17.0	200	36–54
Betamethasone	0.6		300	36–54
Fludrocortisone	2.0	12.0	200	18–36
Desoxycorticosterone acetate			70	

## Data Availability

All authors confirm that all the data that support the findings of this report are available on request from the corresponding author.
